# Cyclic bisphosphonate therapy reduces pain and improves physical functioning in children with osteogenesis imperfecta

**DOI:** 10.1186/s12891-018-2252-y

**Published:** 2018-09-24

**Authors:** Melissa D. Garganta, Sarah S. Jaser, Margot A. Lazow, Jonathan G. Schoenecker, Erin Cobry, Stephen R. Hays, Jill H. Simmons

**Affiliations:** 1 Medical University of South Carolina, Greenville, SC USA; 2 Vanderbilt University Medical Center, Nashville, TN USA; 3 Cincinnati Children’s Hospital Medical Center, Cincinnati, OH USA; 4 Division of Pediatric Endocrinology and Diabetes, Village at Vanderbilt, 1500 21st Avenue South, Suite 1514, Nashville, TN 37212-3157 USA

**Keywords:** Osteogenesis imperfecta, Bisphosphonate, Pamidronate, Zoledronic acid, Pain, Faces®, Quality of life, PedsQL™, Pediatrics

## Abstract

**Background:**

Children with osteogenesis imperfecta (OI) experience pain and impaired physical functioning. The longitudinal effect of cyclic bisphosphonate treatment on these symptoms has not been described. We serially evaluated pain and functioning in pediatric patients with OI treated with intravenous bisphosphonate therapy.

**Methods:**

Pain and physical functioning were assessed at multiple time-points over two infusion cycles in 22 OI patients (median age 10 years [range 2–21 years]; 8 girls) receiving cyclic intravenous bisphosphonate therapy. Pain was assessed using the FACES® visual analogue scale; physical functioning, including self-care, was assessed using the PedsQL™ Generic Core inventory.

**Results:**

Pain scores decreased significantly immediately following infusion and remained reduced at 4 weeks post-infusion, increasing before and decreasing again after subsequent infusion (*F* = 25.00, *p* < 0.001). Physical functioning scaled scores improved 4 weeks after infusion and declined before subsequent infusion across patients (*F* = 10.87, *p* = 0.007). Exploratory analyses indicated significantly different effects between mild and moderate-severe OI types for pain, but not for physical functioning. No fractures occurred during the study.

**Conclusion:**

In children with OI, cyclic intravenous bisphosphonate therapy transiently reduces pain and improves functional abilities. Pain relief occurs immediately following infusion with functional improvements observed 4 weeks later. Both pain and physical functioning return to pretreatment levels by the subsequent infusion.

## Background

Osteogenesis imperfecta (OI) is a genetic disorder that affects type 1 collagen and has variable manifestations including low bone mineral density, skeletal deformities, recurrent bone fractures, scoliosis, and chronic pain [1–3]. The majority of patients with OI have a mutation in the *COL1A1* or *COL1A2* gene encoding the alpha 1 and alpha 2 chains of type 1 collagen, producing either defective type 1 collagen or a deficient quantity of normal type 1 collagen; more than 200 mutations in multiple genes have been identified [4, 5]. The widely accepted Sillence classification distinguishes the four most common phenotypic presentations (Types I-IV) (Table 1), although up to 17 types have been described based upon either unique phenotype or molecular etiology [6–8]. Regardless of type, many patients with OI report pain on a chronic basis, even during periods without fractures [4, 9]. The specific etiology of the pain that occurs in patients with OI in the absence of an acute fracture is not entirely clear; however, it has been hypothesized that inflammatory cytokines, such as prostaglandins or thromboxanes, may contribute to bone turnover and therefore result in pain symptoms [10]. Chronic pain may result in delayed motor development, missed school days and significant psychosocial stress for patients and their families [11, 12]. Furthermore, children with OI experience reduced physical functioning, such as poor mobility and inability to perform routine activities of daily living [2, 13].

**Table 1:** Sillence classification system for osteogenesis imperfecta

OI Type	Features
Type I	Mildest form. Limited bone deformity and fragility.
Type II	Lethal in perinatal period.
Type III	Most severe form in patients who survive the newborn period.
Type IV	Moderate severity. Spectrum of severity between types I and III.
Additional OI types	Range in severity. Etiology is typically due to mutations in genes involved with collagen formation other than *COL1A1* or *COL1A2*.

The current mainstay of pharmacologic treatment for OI is nitrogen-containing bisphosphonate therapy, which has been shown to decrease bone turnover, improve bone mineral density and reduce fracture rates [14, 15]. Bisphosphonate therapy is typically initiated when children with OI have two fragility fractures per year. Although dosing schedules vary between institutions, pamidronate is typically given every 2 months until age 2 years, every 3 months in the third year of life, and every 4 months thereafter; zoledronic acid is generally given every 6 months. The choice of bisphosphonate administered is typically dependent upon provider and patient comfort [16]. It has previously been reported that bisphosphonate therapy relieves bone pain acutely following infusion in children with OI, but a sustained long-term effect, if any, is unclear [2, 3, 14, 17–19]. A recent study reported decreased pain at 1 week post-infusion with zoledronate compared to pre-infusion [20]. Previous studies have demonstrated that cyclic bisphosphonate treatment with pamidronate may improve mobility and muscle force in children with moderate to severe types of OI and may reduce the number of days per week with pain [8], but the effect on severity of pain between infusion cycles has not been well- assessed. Some studies have reported improvement in mobility and independence of activities following bisphosphonate treatment, whereas others have not demonstrated any beneficial effects on physical functioning [2, 3, 13, 21]. Therefore, we assessed the effect of cyclic intravenous bisphosphonate treatment on patient-reported pain levels and parent-assessed physical functioning over time and compared effects between children with mild and moderate-to-severe forms of OI.

## Methods

### Patient population and study design

The sample in this prospective longitudinal cohort study consisted of 22 children with a diagnosis of OI receiving intravenous bisphosphonate therapy at the Monroe Carell Jr. Children’s Hospital at Vanderbilt between November 2014 and February 2016. Patients that had experienced at least 2 fragility fractures within the 12 months prior to starting therapy were included and patients naïve to bisphosphonate treatment were excluded. All patients approached agreed to be enrolled.

OI Type (Type I, Type III or Type IV) was defined clinically, and one patient had genetic testing consistent with OI Type VIII. Patients were followed under real-world conditions through two cycles of bisphosphonate treatment with pain assessed by patient report immediately before and after each infusion and 4 weeks following the initial infusion. Physical functioning was assessed immediately before each infusion and 4 weeks after the first infusion by a parent-completed survey. This study was approved by the Institutional Review Board at Vanderbilt University Medical Center. The authors have no conflicts of interest to disclose.

### Treatments

Patients received either pamidronate or zoledronic acid per their chronic regimen. Pamidronate was administered in a single-day infusion dosed in a weight- and age-dependent manner. Children ages 2–3 years received 1.1 mg/kg every 3 months and those > 3 years received 1.5 mg/kg/dose every 4 months (maximum dose ≤45 mg/infusion and 4.5 mg/kg/year). Zoledronic acid was administered in a single-day infusion of 0.05 mg/kg every 6 months for all ages. The treatment regimen was selected prior to study participation by patients and families after discussion about long-term knowledge of the effects of pamidronate compared with ease of administration of zoledronic acid.

### Assessments

Pain was evaluated using the FACES® visual analogue scale, a validated tool for assessing patient-perceived pain on a scale of 0–10 with 0 indicating no pain and 10 indicating worst possible pain [22]. The specific location of pain was not recorded. Physical functioning was assessed using the Pediatric Quality of Life Inventory 4.0 Generic Core Scales for Physical Functioning (PedsQL™) Parent Reports for Young Children ages 5–7, Children ages 8–12 and Teens ages 13–17 years. The PedsQL™ is a health-related quality of life measure that has demonstrated validity and reliability in a wide variety of general populations and in several disease-specific pediatric populations [23–25]. The Physical Functioning Core Scale consists of eight items related to daily activities specific to age group (e.g., walking, running, bathing, lifting items, energy level). Physical Functioning Core Scale score is calculated as a scaled score ranging from 0 to 100, with 100 indicating the highest level of health-related quality of life and physical functioning [25].

### Statistical analyses

Descriptive analyses were conducted to determine the means and standard deviations of the measures at each time point. To assess changes in pain and physical functioning over time (within subjects), we conducted repeated measures analysis of variance. The repeated measures design provides greater statistical power to detect effects with a smaller number of patients by controlling for individual differences. We also conducted exploratory analyses to determine whether there were differences in changes in pain across OI types (Type I vs. Types III and IV). Analyses were performed using IBM SPSS software, version 23 (SPSS, Chicago, IL).

## Results

Clinical characteristics of the 22 children analyzed in this study are presented in Table 2. Patients were 2–21 years old (median age 10 years); 36% had mild OI (Type I, *N* = 8) while 64% had moderate-severe OI (Type III, *N* = 7; Type IV, *N* = 6; Type VIII, *N* = 1), as defined by clinical characteristics (Sillence classification (Table 1)), except for the patient with Type VIII, who was classified based upon genetic testing (moderate-severe). Not all patients were able to complete all study visits, though this was never due to patient/parent refusal. As these patients were not seen under specific study conditions, but rather were followed at their regularly scheduled appointments, some patients missed study windows due to cancellations, rescheduling or no- shows to their appointments, or research staff being unavailable during their appointment times. The median time between Visit 1 (initial infusion during study) and Visit 2 (per protocol, 4 weeks post infusion) was 30.5 days. The median time between Visit 1 (initial infusion during study) and Visit 3 (2nd infusion) was 206 days (6.9 months). Overall, 73% (*N* = 16) of patients were treated with pamidronate, while 27% (*N* = 6) were treated with zoledronic acid. There were no significant differences between patients treated with pamidronate and zolendronic with respect to child age, sex, or OI Type. No fractures occurred during the study.

**Table 2:** Clinical characteristics of the study population

Characteristic	*N* = 22
Age (years) ^a^	10 (2–21)
Male/Female	14/8 (64%/36%)
White	14 (64%)
Black	4 (18%)
Asian	2 (9%)
Latino	1 (5%)
Multiracial	1 (5%)
OI Type I	8 (36%)
OI Type III	7 (32%)
OI Type IV	6 (27%)
OI Type VIII	1 (5%)
Height z-score ^a^	− 2.96 (− 12.43–4.16)
Weight z-score ^a^	− 1.64 (− 11.89–3.29)
BMI z-score ^a^	0.81 (− 0.95–2.51)
Able to ambulate without assistance	14/21 (67%)
Receiving pamidronate	16 (73%)
Receiving zoledronic acid	6 (27%)

^a^ Median (range)

### Pain

Eighteen patients completed the pre- and immediately post- infusion pain assessments, 12 patients completed the 4-week post-infusion assessment, and eight patients completed the subsequent pre-infusion pain assessment. One patient sustained acute trauma by falling from a chair during his infusion and his pain scores were excluded.

**Fig. 1:**
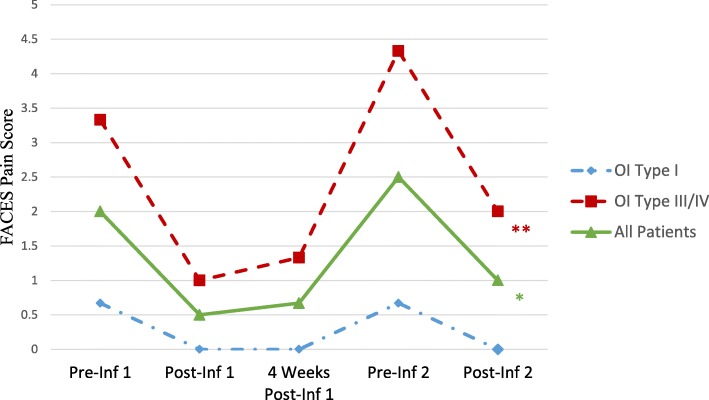
Mean pain scores over time by osteogenesis imperfecta (OI) type, assessed by FACES® visual analogue scale immediately pre- / immediately post-infusion, 4 weeks post-infusion, and immediately pre- / immediately post subsequent infusion. Scale reduced (maximum pain score 5) for visual effect. * *P* < 0.001 for effect over time in all patients. ** *P* = 0.023 for difference in pain over time between patients with milder (Type I) and more severe (Types III/IV) OI

For patients who had data for all time points (*n* = 6), there was a significant change in self-reported pain scores over time associated with bisphosphonate infusion (*F* = 10.19, *P* < 0.001) (Fig. 1). Pain scores decreased from 2.00 ± 2.00 immediately pre-infusion to 0.50 ± 0.84 immediately post-infusion. Pain scores remained decreased from baseline at 4 weeks post-infusion, with a mean score of 0.67 ± 0.82. A diminished analgesia was observed with pain scores returning to pre-infusion levels (2.50 ± 2.43) immediately prior to subsequent infusion, approximately 6 months later. Significant analgesia was again noted immediately following subsequent infusion (pain scores 1.00 ± 1.27). For the 12 patients who had pain assessments only at the first pre- and post- infusion and 4-week follow-up visit, there was a significant change in self-reported pain scores from the first infusion to the follow-up visit, about 4 weeks later (*F* = 25.00, *P* < .001). Pain scores decreased from 2.17 ± 1.53 prior to infusion to 0.42 ± 0.79 post-infusion and remained decreased from baseline at 4 weeks post-infusion, with a mean score of 0.50 ± 0.67. It is notable that there was a significant difference between pre-infusion pain scores at the first visit and follow-up visit (*t* = −.2.65, *P* = 0.033).

Exploratory subgroup analysis indicated that the effect of bisphosphonate infusion on pain scores differed according to type of OI (Fig. 1). We identified a significant Time x OI Type interaction effect for pain (*F* = 3.82, *P* = 0.023): children with more severe forms of OI (Types III/IV, *n* = 3) reported significantly higher levels of pain at baseline, and a greater decrease in pain following bisphosphonate infusion, than children with milder OI (Type I, *n* = 3). In patients with Types III/ IV OI, mean pain scores decreased from 3.33 ± 2.08 pre-infusion to 1.00 ± 1.00 post-infusion, and remained decreased at 1.33 ± 0.58 4 weeks post-infusion. In patients with Type I OI, mean pain scores decreased from 0.67 ± 0.58 pre-infusion to 0.00 ± 0.00 post-infusion, and remained decreased at 0.00 ± 0.00 4 weeks post- infusion. A similar pattern was seen with the second infusion cycle. Children with more severe OI (Types III/IV) reported mean pain scores of 4.33 ± 2.08 prior to infusion, decreasing to 2.00 ± 1.00 immediately after the second infusion; children with milder OI (Type I) reported mean pain scores of 0.67 ± 0.58 pre-infusion, decreasing to 0.00 ± 0.00 immediately after infusion. These results must be interpreted with caution and are therefore considered exploratory; given the observed effect size and our sample of six participants measured across five time points, we had power of .24 to detect a significant effect. A sample size of 16 (8 per group) was needed to have sufficient power to detect significant effects.

### Physical functioning

Parents of all 22 patients completed the pre-infusion physical functioning assessment, 12 parents completed the 4-week post-infusion assessment, and seven parents completed the subsequent pre-infusion physical functioning assessment.

**Fig. 2:**
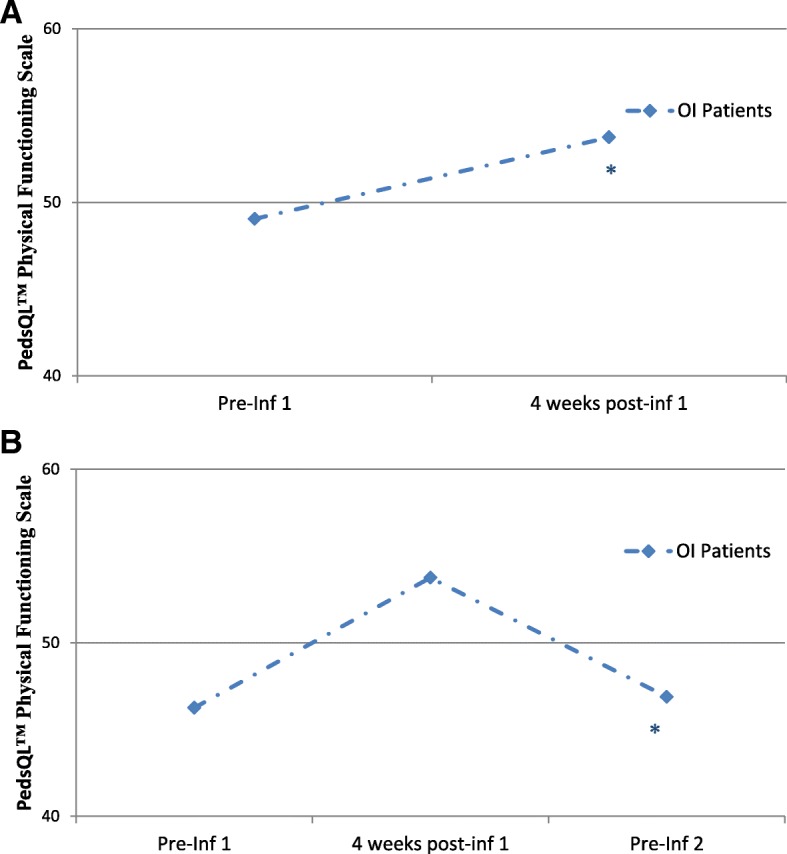
**a** Mean physical functioning scaled scores over time, assessed using the PedsQL™ Generic Core Scale for Physical Functioning pre-infusion and 4 weeks post-infusion (*n* = 12). * *P* = 0.007.**b** Mean physical functioning scaled scores over time, assessed using the PedsQL™ Generic Core Scale for Physical Functioning pre-infusion, 4 weeks post-infusion, and pre-subsequent infusion (*n* = 5). * P = 0.008. Scale reduced (minimum scaled score 40) for visual effect

For the patients who had data for only the first two visits (*n* = 12, Fig. 2a), we observed significant change from the first visit to the post-visit assessment in parent-reported physical functioning scaled scores associated with bisphosphonate infusion (*F* = 10.87, *P* = .007). Mean scaled score for physical functioning was 49.48 ± 25.49 before infusion, and increased to 57.03 ± 25.29 4 weeks after infusion.

For the patients who had data from all time points (*n* = 5, Fig. 2b), we observed a significant change in parent-reported physical functioning scaled scores. This effect was quadratic (*F* = 24.61, *P* = .008), such that physical functioning improved after the first infusion, and diminished to pre-infusion levels by Visit 3. Specifically, mean scaled score for physical functioning was 46.25 ± 31.59 before infusion, increased to 53.75 ± 29.60 4weeks after infusion, and returned to 46.88 ± 28.13 by the second infusion cycle. We were not able to evaluate differences in physical function between OI types due to small sample size.

## Discussion

We demonstrate that treatment with cyclic bisphosphonate infusions in a real-world setting reduces pain and improves reported physical functioning in children with both mild and moderate-severe OI. Patients experience pain relief immediately following infusion, with sustained analgesia for several weeks. This effect eventually wanes over time, with pain returning at least to pre-infusion baseline prior to the next infusion. The children in the current study also experience a similar improvement in parental reports of physical functioning, including the ability to participate in exercise, play, household chores and self-care activities, such as bathing, following infusions, but these gains appeared to decrease back to baseline prior to the next cycle.

Our findings are consistent with the results of previous studies investigating the short-term effect of bisphosphonate infusions on pain relief in children with OI, in which consecutive three-day infusions reduced the number of days with pain per week in the weeks following the infusion, with recurrence of pain preceding the next treatment cycle [14, 17]. A recent study which evaluated pain in patients who were administered zoledronic acid in the hospital setting similarly showed reduction in both pain scores and number of pain quality descriptors measured following infusions, but neither were statistically significant [20]. Similar to our findings, these aforementioned studies also demonstrated improvements in physical functioning, notably mobility and ambulation, following bisphosphonate infusions, with decline in functioning prior at the time of the subsequent infusion [14, 17, 20]. However, these studies were not performed in patients receiving outpatient single-day infusions of bisphosphonate therapy [26]. Our results indicate that reduced pain and improved physical functioning can be achieved using single-day outpatient treatment, which is less cumbersome for patients and families than the traditional consecutive three-day infusion cycle. Other potential areas of investigation include determining if these effects on pain and functioning can be achieved with oral bisphosphonate therapy.

The results of the current study contribute to growing evidence supporting temporary gains in pain relief and physical functioning following bisphosphonate infusions, but it remains unclear if and how these improvements after bisphosphonate use can be sustained long-term. Prior studies did not demonstrate significant long-term improvements in pain, mobility, or functioning at time points of 12 months or more following administration of bisphosphonates [2, 3, 18, 19], though two of these trials utilized different bisphosphonate types (risedronate and alendronate) than the ones administered in this study. Further research investigating the optimal bisphosphonate cycle duration and type as well as supplementary therapies to ensure sustained pain and functional improvements is therefore needed.

As expected, children with more severe forms of OI have more pain at baseline than children with milder forms of OI. While exploratory, our findings suggest that more severely affected OI patients have greater improvement in pain scores after infusions, while patients with milder OI experience less dramatic pain relief. Why patients with more severe forms of OI should obtain greater pain relief following bisphosphonate infusion than patients with milder OI is unclear. Additionally, the mechanism through which bisphosphonate treatment provides pain relief is unclear. Previous studies have suggested that decreased rates of fracture and bone turnover may provide relief from pain, but this does not explain the significant analgesia reported immediately following a single-day infusion as demonstrated in this study. Reduction of inflammatory cytokines such as serum prostaglandins or thromboxanes may play a role, as suggested by previous studies [10]. Future studies should include objective laboratory data such as measurement of the inflammatory cytokine prostaglandin E2, which has been implicated as a potent contributor to bone remodeling [10, 27], correlated with subjective measures of pain and functioning.

In the current cohort, patients with mild OI had experienced fractures prior to initiation of treatment with bisphosphonate therapy, as fragility fractures are typically considered a requirement for treatment. Further studies are needed to examine the effect of bisphosphonate therapy on pain, physical functioning and quality of life in patients with mild forms of OI who do not have fragility fractures.

Limitations of this study include small sample size and lack of either a placebo-controlled or non-treatment control group. The small sample size provided insufficient statistical power to evaluate for racial or gender differences in pain and functioning, or to determine differences between the two bisphosphonates used in this cohort. Patients were on chronic bisphosphonate therapy, so we were unable to evaluate the effect of first bisphosphonate infusion, as patients undergoing their first bisphosphonate infusion were excluded due to the flu-like reaction that often occurs in this setting. It would be appropriate in other investigations to evaluate effects on pain and physical functioning of first bisphosphonate infusion after this flu-like reaction has resolved. Additionally, the patient sample size was too small to determine whether there is a cumulative effect of bisphosphonate therapy on pain and physical functioning. The time between infusions was a mean of 6 months, making it difficult to determine exactly when the pain began to increase again following the initial infusion. In addition, while the timing between infusions varies depending on the type of bisphosphonate administered (zoledronate vs pamidronate), the current study did not have a large enough population to evaluate whether there was a significant difference in pain relief or improved functioning between these two groups. However, understanding both the exact time at which pain starts to worsen as well as whether analgesia is impacted by bisphosphonate type could be important in determining the duration and type of therapy which maximally reduces pain and improves physical functioning in these patients. Additionally, the exact locations of pain were not assessed, but would be useful to identify in future studies, in order to more specifically evaluate the impact of bisphosphonates. Physical functioning was not assessed by medical provider evaluation, but by parental survey. The PedsQL™ is an inventory not approved specifically for OI, but has been used in other chronic pediatric disease-specific populations [28, 29]. The FACES® scale used to evaluate pain is not specific for a population with OI; unfortunately, there is no pain assessment available specifically for children and adolescents with OI [30].

## Conclusions

Our data indicate that chronic cyclic treatment with single-day bisphosphonate infusions acutely reduces pain and improves parental reports of daily functioning, including ability to participate in self-care, in children with OI regardless of type. Effects on pain appear to differ according to type of OI. Pain relief occurs immediately following infusion with functional improvements observed 4 weeks later. Both pain and physical functioning return to pretreatment levels by the subsequent infusion.

## Abbreviations

OI: osteogenesis imperfecta
PedsQL™: Pediatric Quality of Life Inventory 4.0 Generic Core Scales for Physical Functioning

## References

[1] Ben Amor M, Rauch F, Monti E, Antoniazzi F (2013). Osteogenesis imperfecta. Pediatr Endocrinol Rev.

[2] Land C, Rauch F, Montpetit K, Ruck-Gibis J, Glorieux FH (2006). Effect of intravenous pamidronate therapy on functional abilities and level of ambulation in children with osteogenesis imperfecta. J Pediatr.

[3] Letocha AD, Cintas HL, Troendle JF, Reynolds JC, Cann CE, Chernoff EJ (2005). Controlled trial of pamidronate in children with types III and IV osteogenesis imperfecta confirms vertebral gains but not short-term functional improvement. J Bone Miner Res.

[4] Ben Amor IM, Glorieux FH, Rauch F (2011). Genotype-phenotype correlations in autosomal dominant osteogenesis imperfecta. J Osteoporos.

[5] Rauch F, Glorieux FH (2004). Osteogenesis imperfecta. Lancet.

[6] Van Dijk FS, Sillence DO (2014). Osteogenesis imperfecta: clinical diagnosis, nomenclature and severity assessment. Am J Med Genet A.

[7] Symoens S, Malfait F (2013). D’hondt S, Callewaert B, Dheedene a, Steyaert W, et al. deficiency for the ER-stress transducer OASIS causes severe recessive osteogenesis imperfecta in humans. Orphanet J Rare Dis.

[8] Mendoza-Londono R, Fahiminiya S, Majewski J, Tétreault M, Nadaf J, Kannu P (2015). Recessive osteogenesis imperfecta caused by missense mutations in SPARC. Am J Hum Genet.

[9] Zack P, Franck L, Devile C, Clark C (2005). Fracture and non-fracture pain in children with osteogenesis imperfecta. Acta Paediatr.

[10] D'Eufemia P, Finocchiaro R, Celli M, Zambrano A, Tetti M, Villani C (2008). High levels of serum prostaglandin E2 in children with osteogenesis imperfecta are reduced by neridronate treatment. Pediatr Res.

[11] Cole DE (1993). Psychosocial aspects of osteogenesis imperfecta: an update. Am J Med Genet.

[12] Huguet A, Miro J (2008). The severity of chronic pediatric pain: an epidemiological study. J Pain.

[13] Sousa T, Bompadre V, White KK (2014). Musculoskeletal functional outcomes in children with osteogenesis imperfecta: associations with disease severity and pamidronate therapy. J Pediatr Orthop.

[14] Forin V, Arabi A, Guigonis V, Filipe G, Bensman A, Roux C (2005). Benefits of pamidronate in children with osteogenesis imperfecta: an open prospective study. Joint Bone Spine.

[15] Devogelaer JP, Coppin C (2006). Osteogenesis imperfecta : current treatment options and future prospects. Treat Endocrinol.

[16] Palomo T, Fassier F, Ouellet J, Sato A, Montpetit K, Glorieux FH (2015). Intravenous bisphosphonate therapy of young children with osteogenesis imperfecta: skeletal findings during follow up throughout the growing years. J Bone Miner Res.

[17] Glorieux FH, Bishop NJ, Plotkin H, Chabot G, Lanoue G, Travers R (1998). Cyclic administration of pamidronate in children with severe osteogenesis imperfecta. N Engl J Med.

[18] Ward LM, Rauch F, Whyte MP, D'Astous J, Gates PE, Grogan D (2011). Alendronate for the treatment of pediatric osteogenesis imperfecta: a randomized placebo-controlled study. J Clin Endocrinol Metab.

[19] Rauch F, Munns CF, Land C, Cheung M, Glorieux FH (2009). Risedronate in the treatment of mild pediatric osteogenesis imperfecta: a randomized placebo-controlled study. J Bone Miner Res.

[20] Tsimicalis A, Boitor M, Ferland CE, Rauch F, Le May S, Carrier JI (2018). Pain and quality of life of children and adolescents with osteogenesis imperfecta over a bisphosphonate treatment cycle. Eur J Pediatr.

[21] Sakkers R, Kok D, Engelbert R, van Dongen A, Jansen M, Pruijs H (2004). Skeletal effects and functional outcome with olpadronate in children with osteogenesis imperfecta: a 2-year randomised placebo-controlled study. Lancet.

[22] Tomlinson D, von Baeyer CL, Stinson JN, Sung L (2010). A systematic review of faces scales for the self-report of pain intensity in children. Pediatrics.

[23] Varni JW, Seid M, Kurtin PS (2001). PedsQL 4.0: reliability and validity of the pediatric quality of life inventory version 4.0 generic core scales in healthy and patient populations. Med Care.

[24] Varni JW, Burwinkle TM, Seid M, Skarr D (2003). The PedsQL 4.0 as a pediatric population health measure: feasibility, reliability, and validity. Ambul Pediatr.

[25] Varni JW, Seid M, Rode CA (1999). The PedsQL: measurement model for the pediatric quality of life inventory. Med Care.

[26] Steelman J, Zeitler P (2003). Treatment of symptomatic pediatric osteoporosis with cyclic single-day intravenous pamidronate infusions. J Pediatr.

[27] Liu XH, Kirschenbaum A, Yao S, Levine AC (2005). Cross-talk between the interleukin-6 and prostaglandin E(2) signaling systems results in enhancement of osteoclastogenesis through effects on the osteoprotegerin/receptor activator of nuclear factor-{kappa}B (RANK) ligand/RANK system. Endocrinology.

[28] Engsberg JR, Ross SA, Collins DR (2006). Increasing ankle strength to improve gait and function in children with cerebral palsy: a pilot study. Pediatr Phys Ther.

[29] Bayle-Iniguez X, Audouin-Pajot C, Sales de Gauzy J, Munzer C, Murgier J, Accadbled F (2015). Complex regional pain syndrome type I in children. Clinical description and quality of life. Orthop Traumatol Surg Res.

[30] Nghiem T, Louli J, Treherne SC, Anderson CE, Tsimicalis A, Lalloo C (2017). Pain experiences of children and adolescents with osteogenesis imperfecta: an integrative review. Clin J Pain.

